# Suprascapular Nerve Pulsed Radiofrequency as an Effective Pain Relief Strategy in Supraspinatus Muscle Tendon Tears

**DOI:** 10.7759/cureus.46936

**Published:** 2023-10-13

**Authors:** Miguel De Castro Correia, Luís Oliveira, Eugénio Moita Gonçalves, Rodrigo Correia, Inês Andrade, Andre Borges, Tiago Rodrigues Lopes, José Luís Carvalho

**Affiliations:** 1 Physical Medicine and Rehabilitation, North Rehabilitation Center, Vila Nova de Gaia, PRT; 2 Physical Medicine and Rehabilitation, Vila Nova de Gaia/Espinho Hospital Centre, Vila Nova de Gaia, PRT; 3 Physical Medicine and Rehabilitation, Alcoitão Rehabilitation Medicine Center, Lisbon, PRT; 4 Physical Medicine and Rehabilitation, Trás-os-Montes E Alto Douro Hospital Center, Vila Real, PRT; 5 Physical Medicine and Rehabilitation, Espregueira-Mendes Sports Center, Porto, PRT

**Keywords:** shoulder rehabilitation, supraspinatus tear, suprascapular nerve pulsed radiofrequency, rotator cuff tear, pain

## Abstract

Introduction: The supraspinatus muscle tendon is the most frequently rotator cuff muscle torn. Reliable shoulder pain relief strategies are needed for patients with severe pain, refractory to conservative management, and without surgical indication. Materials and methods: We conducted a retrospective analysis in a Portuguese reference Rehabilitation Centre during the 1st of January 2020 and the 30th of June 2021, including all patients with partial or complete supraspinatus tendon tear, older than 50 years, who presented with severe pain and who were submitted to suprascapular nerve pulsed radiofrequency. Results: We included 32 patients in our retrospective analysis, mainly female (53%) with a mean age of 66.50 years old. Most of the patients reported right shoulder pain (21 patients, 66%). The mean baseline pain, reported on the numeric rating scale, was 8.00 ± 0.88. Compared to baseline, mean pain reduced 4.00 ± 3.19 at three months (p<0.001), 3.59 ± 3.13 at six months (p<0.001) and 2.94 ± 2.78 at 12 months (p<0.001). From the 3rd to the 12th month there was an increase of 1.06 ± 2.77 in mean pain (p=0.038). There was no difference (p>0.05) in average pain at 0, 3, 6, or 12 months between patients who were simultaneously submitted to an intra-articular shoulder injection. Discussion: Our analysis revealed 36% average shoulder pain reduction for, at least, 12 months following suprascapular nerve pulsed radiofrequency, with a peak pain reduction of 50% at three months. Slow fading of pain reduction in the following nine months was seen, however, compared to baseline, pain reduction was always statistically significant. Cortico-anaesthetic intra-articular shoulder injections seem not to add benefit in shoulder pain reduction when performed simultaneously with suprascapular nerve pulsed radiofrequency. Conclusion: Suprascapular nerve pulsed radiofrequency seems to be an effective strategy for shoulder pain reduction, in patients with severe pain, refractory to other management modalities. Nonetheless, larger prospective studies, analyzing shoulder functionality and quality of life lost scores, besides pain reduction, should be pursued.

## Introduction

The shoulder complex comprises three true joints - sternoclavicular (SC), acromioclavicular (AC) and glenohumeral (GH) joints - and a false one - scapulothoracic joint [1]. The GH joint is formed between the convex humerus head and the scapular glenoid fossa [1]. Functionally, this joint presents as one of the most important joints of the shoulder complex, as it is responsible for two-thirds of the scapulohumeral abduction rhythm [2]. In addition to static stabilizers (such as the capsule and ligaments), the GH joint is dynamically reinforced (although not exclusively) by the rotator cuff muscles: subscapularis, supraspinatus, infraspinatus and teres minor [3]. These four muscles originate in different anatomic features of the scapula and insert either at the lesser tubercle (subscapularis) or at the greater tubercle [1]. Their detailed function is complex and out of this paper's scope. Nonetheless, on a simple GH joint approach, subscapularis is an internal rotator, supraspinatus is an abductor, and infraspinatus and teres minor are external rotators [4].

Probably due to the functional relevance of the rotator cuff muscles, clinical relevance arises as well, with rotator cuff injuries being the most common cause of disability related to the shoulder [5]. The term ‘rotator cuff injury’ is a broad spectrum beginning at tendinopathy and ending at complete tears. Due to its degenerative progression, age is the most common factor for rotator cuff disease [6]. Other risk factors have also been identified, including smoking, family history, poor posture, trauma, hypercholesterolemia, and occupation [7,8]. Patients with rotator cuff tears usually present with shoulder pain and limited function [9]. Some authors describe partial tears as more painful than full-thickness tears [10]. Although ultrasonography is widely used in clinical practice, shoulder MRI remains the diagnosis gold standard [9].

The supraspinatus muscle is the most frequently rotator cuff muscle torn [11]. Pain from a supraspinatus muscle tear is transmitted via the suprascapular nerve [12]. These injuries are usually managed conservatively with a proper rehabilitation program focused on decreasing pain, increasing range of motion and muscle strengthening [9]. However, even after adequate conservative management, patients might still experience severe pain. These patients may continue not to have a formal surgical indication or may not want to be surgically managed. In these cases, the need for a reliable pain relief strategy arises.

Pulsed radiofrequency (pRF) uses radiofrequency in short (20ms) high-voltage bursts with an OFF phase of 480ms, allowing heat to be eliminated [13]. In part, pRF arose due to the need of developing a less destructive alternative to continuous radiofrequency (cRF) [13]. cRF has been used to treat pain since 1974 [14], as opposed to pRF which has gained more scientific relevance in the past few years. Although the mechanism by which pRF causes pain relief isn’t consensual, studies suggest molecular pathways, involving the *c-Fos* gene (an immediate early gene) [15] or ATF3 (a cellular stress indicator) [16], which affect neuronal membrane and, thus, pain signaling.

The aim of this study was to analyze the pain relief magnitude of suprascapular nerve pulsed radiofrequency (SNpRF) in patients with painful supraspinatus muscle tendon tears, refractory to conservative management.

## Materials and methods

We conducted a retrospective analysis of a Musculoskeletal Rehabilitation and Intervention Unit external consultation reports of a Portuguese reference Rehabilitation Centre, during the 1st of January 2020 and the 30th of June 2021. We included all patients with partial or complete supraspinatus tendon tears (either with or without other rotator cuff tendon tears), older than 50 years, who presented with severe pain (numerical rating scale (NRS) equal to or bigger than 7 out of 10) and who were submitted to suprascapular nerve pulsed radiofrequency (2 x 2 minutes, 42ºC, ON: OFF ratio of 20: 480ms). Literature is teeming with rotator cuff tears classification [17], so the need to define rotator cuff tears arose. We considered complete supraspinatus tendon tears as full-thickness tears with complete anterior to posterior involvement. Every other tear was considered a partial tear. Regarding exclusion criteria, patients whose rotator cuff tear was previously surgically managed, and patients with central nervous system diseases that might lead to pain (namely, a spinal cord injury and a post-poliomyelitis patient) were excluded. A total of 39 patients were initially included. We consulted consultation clinical records at three, six and 12 months after the procedure (when information was missing the patients were contacted by phone call). After re-evaluation, seven patients were excluded due to partial tear progression to complete tear, with consequent surgical management. The major outcome was a reduction in pain by a numerical rating scale. The statistical analysis was made with IBM SPSS Statistics version 27.0 (IBM Corp., Armonk, NY). An independent samples test was used to compare basal pain NRS between groups (full sample; patients only submitted to pulsed radiofrequency; and patients submitted to pulsed radiofrequency and intra-articular cortico-anesthetic injection). Levene's test was used to ensure equal variance between groups. We used a non-parametric test (Wilcoxon signed-rank test) to compare NRS between 0, three, six and 12 months in each group.

## Results

Sample characterization

After re-evaluation at three, six and 12 months, we included 32 patients in our retrospective analysis. Of these, 17 (53%) were female and 15 (47%) were male. The mean age was 66.50 years ± 8.88 years. Most of the patients reported right shoulder pain (21 patients, 66%). As stated, all our patients had a supraspinatus muscle tear, with 11 being complete tears (34%) and 21 being partial tears (66%). Additionally, 17 patients (53%) had other rotator cuff muscles torn. Table 1 describes partial and complete rotator cuff muscle tear prevalence in this population.

**Table 1:** Partial and complete rotator cuff tears prevalence in our sample We considered complete tears as full-thickness tears with complete antero-posterior extension; every other tear was considered a partial tear.

Muscle Tendon	No injury described (n/%)	Partial Tear (n/%)	Complete Tear (n/%)
				Supraspinatus	0 / 0%	21 / 66%	11 / 34%
Infraspinatus	16 / 50%	14 / 44%	2 / 6%
Teres minor	30 / 94%	1 / 3%	1 / 3%
Subscapularis muscle	26 / 81%	6 / 19%	0 / 0%

Analgesic drugs consumption

Regarding daily scheduled analgesic drugs, 21 (66%) clinical records didn’t specify whether patients were or weren’t previously prescribed any pain medication. On the other hand, four patients (13%) reported using a daily medication, three of which were opioids (tramadol, tapentadol or hydromorphone). The remaining seven patients (22%) didn’t have any daily analgesic drug. About on-demand analgesic drugs, once again, most of the clinical records (n=18, 56%) didn’t specify whether the patients had or hadn’t any drug prescribed. Six patients (19%) denied any on-demand medication and eight patients (25%) reported its use, four used nonsteroidal anti-inflammatory agents (NSAIDs) (naproxen, etodolac or nimesulide) and the other four used opioids (tramadol, tapentadol or buprenorphine). Most patients (n=19, 59%) were previously submitted to intra-articular ultrasound-guided injections (one hyaluronic acid injection and 18 cortico-anaesthetic injections).

Pain comparison: full sample

Considering our whole sample, the mean baseline pain, reported on the numeric rating scale, was 8.00 ± 0.88. Compared to baseline, mean pain reduced 4.00 ± 3.19 at three months (p<0.001), 3.59 ± 3.13 at six months (p<0.001) and 2.94 ± 2.78 at 12 months (p<0.001). As reported in Table 2, no statistically significant difference was found between mean pain at three and six months (p=0.350), nor between pain at six and 12 months (p=0.105). Average pain increased by 1.06 ± 2.77 (p=0.038) between three and 12 months.

**Table 2:** Full sample average pain comparison between different evaluation periods The first column indicates the beginning (X) and the end of the same period (Y). The second and third columns show the mean and standard deviation of pain in periods X and Y, respectively. The fourth column shows pain variation between periods X and Y. The fifth column represents the statistical difference (p-value).

Full Sample n=32 (period X to Y)	Average Pain X	Average Pain Y	Variation (Y-X)	p-value
					0 to 3 months	8.00 ± 0.88	4.00 ± 3.16	- 4.00 ± 3.19	<0.001
0 to 6 months	8.00 ± 0.88	4.41 ± 3.12	- 3.59 ± 3.13	<0.001
0 to 12 months	8.00 ± 0.88	5.06 ± 2.81	- 2.94 ± 2.78	<0.001
3 to 6 months	4.00 ± 3.16	4.41 ± 3.12	+ 0.41 ± 2.42	0.350
3 to 12 months	4.00 ± 3.16	5.06 ± 2.81	+ 1.06 ± 2.77	0.038
6 to 12 months	4.41 ± 3.12	5.06 ± 2.81	+ 0.66 ± 2.22	0.105

**Figure 1:**
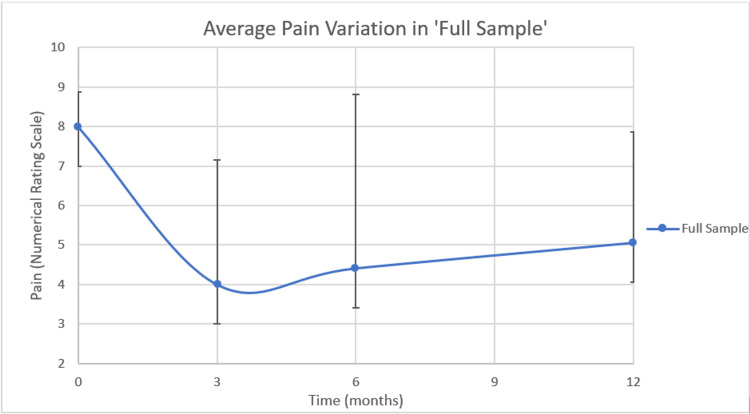
Average pain variation in the full sample

Figure 1 shows variation between average pain at baseline, three months, six months, and 12 months in ‘Full Sample’.

Pain reduction: pulsed radiofrequency vs pulsed radiofrequency and intra-articular injection

All 32 patients were submitted to ultrasound-guided suprascapular nerve pulsed radiofrequency (two 2-minute cycles, 42ºC, ON: OFF ratio of 20: 480ms). Simultaneously, 12 patients (37.5%) were also submitted to an intra-articular cortico-anaesthetic ultrasound-guided cortico-anaesthetic injection (80mg of methylprednisolone and 30mg of lidocaine). The mean baseline pain in the latter group was 7.83 ± 0.72, compared to 8.10 ± 0.97 in the isolated pulsed radiofrequency group (p=0.415). Moreover, as stated in Table 3, comparing average pain at three, six and 12 months between isolated pulsed radiofrequency patients (‘pRF’) and pulsed radiofrequency plus intra-articular injection (‘pRF + IA’) patients, there was no statistically significant difference.

**Table 3:** Average pain comparison at 0, three, six and 12 months between 'pRF' and 'pRF + IA' patients pRF - patients who were only submitted to pulsed radiofrequency; pRF + IA - patients who were simultaneously submitted to pulsed radiofrequency and intra-articular injection

Period	Average Pain ’pRF’ n=20	Average Pain ’pRF + IA’ n=12	p-value
				0 months	8.10 ± 0.97	7.83 ± 0.72	0.415
3 months	4.00 ± 3.31	4.00 ± 3.04	1
6 months	4.00 ± 3.21	5.08 ± 2.97	0.35
12 months	4.55 ± 2.98	5.92 ± 2.39	0.188

Table 4 shows the average pain variation in the ‘pRF only’ and Table 5 in the ‘pRF + IA’ group. Considering the pulsed radiofrequency alone group, average pain reduction compared to baseline was 4.10 ± 3.31 (<0.01) at three months, 4.10 ± 3.23 at six months (p<0.001) and 3.55 ± 2.91 at 12 months (p<0.01). In this group, there was no statistically significant difference between average pain at three and six months, neither between three and 12 months nor between six and 12 months. Regarding the group of patients who were submitted to pulsed radiofrequency and intra-articular injection, average pain reduction compared to baseline was 3.83 ± 3.13 (p=0.001) at three months, 2.75 ± 2.90 at six months (p=0.07) and 1.92 ± 2.31 at 12 months (p=0.015). Differently from the previous group, there was a significant statistical increase between average pain at three and six months, as well as three and 12 months. As in the ‘pRF’ group, no statistically significant difference was found between six and 12 months.

**Table 4:** Average pain comparison between different evaluation periods in the 'pRF' group The first column indicates the beginning (X) and the end of the same period (Y). The second and third columns show the mean and standard deviation of pain in periods X and Y, respectively. The fourth column shows pain variation between periods X and Y. The fifth column represents the statistical difference (p-value). pRF - patients who were only submitted to pulsed radiofrequency.

‘pRF’ Group n=20 (period X to Y)	Average Pain X	Average Pain Y	Variation (Y-X)	p-value
					0 to 3 months	8.10 ± 0.97	4.00 ± 3.31	- 4.10 ± 3.31	<0.001
0 to 6 months	8.10 ± 0.97	4.00 ± 3.21	- 4.10 ± 3.23	<0.001
0 to 12 months	8.10 ± 0.97	4.55 ± 2.98	- 3.55 ± 2.91	<0.001
3 to 6 months	4.00 ± 3.31	4.00 ± 3.21	0.00 ± 2.74	1
3 to 12 months	4.00 ± 3.31	4.55 ± 2.98	+ 0.55 ± 2.86	0.400
6 to 12 months	4.00 ± 3.21	4.55 ± 2.98	+ 0.55 ± 2.21	0.280

**Table 5:** Average pain comparison between different evaluation periods in the 'pRF + IA' group The first column indicates the beginning (X) and the end of the same period (Y). The second and third columns show the mean and standard deviation of pain in periods X and Y, respectively. The fourth column shows pain variation between periods X and Y. The fifth column represents the statistical difference (p-value). pRF + IA - patients who were simultaneously submitted to pulsed radiofrequency and intra-articular injection.

‘pRF + IA’ Group n=12 (period X to Y)	Average Pain X	Average Pain Y	Variation (Y-X)	p-value
					0 to 3 months	7.83 ± 0.72	4.00 ± 3.04	- 3.83 ± 3.13	0.001
0 to 6 months	7.83 ± 0.72	5.08 ± 2.97	- 2.75 ± 2.90	0.007
0 to 12 months	7.83 ± 0.72	5.92 ± 2.39	- 1.92 ± 2.31	0.015
3 to 6 months	4.00 ± 3.04	5.08 ± 2.97	+ 1.08 ± 1.68	0.047
3 to 12 months	4.00 ± 3.04	5.92 ± 2.39	+ 1.92 ± 2.50	0.022
6 to 12 months	5.08 ± 2.97	5.92 ± 2.39	+ 0.83 ± 2.33	0.241

**Figure 2:**
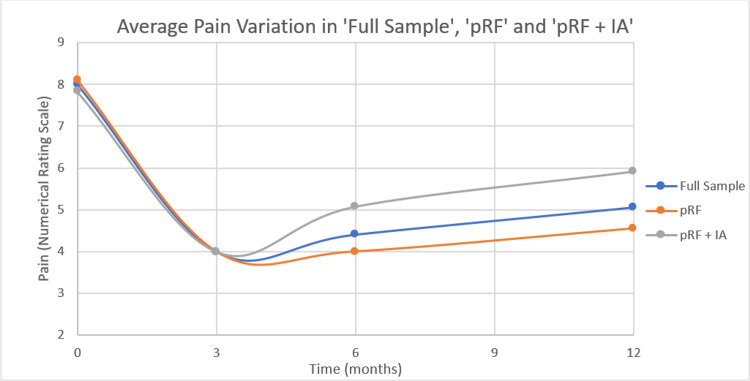
Average Pain Variation in ‘Full Sample’, ‘pRF’ and ‘pRF + IA’ groups pRF - patients who were only submitted to pulsed radiofrequency; pRF + IA - patients who were simultaneously submitted to pulsed radiofrequency and intra-articular injection

Figure 2 graphically shows variation between average pain at baseline, three months, six months, and 12 months in both the ‘pRF’ and ‘pRF + IA’ groups, as well as in the ‘Full Sample’.

Pain reduction: partial vs complete supraspinatus muscle tears

As referred to in Table 1, most of our patients had a partial supraspinatus tear (n=21). We compared average pain at baseline, three months, six months, and 12 months in patients with partial and complete tears. The results are shown in Table 6, without any statistically significant difference between groups.

**Table 6:** Average pain comparison at 0, three, six and 12 months between partial supraspinatus muscle tear patients and complete supraspinatus muscle tear patients

Period	Average Pain Partial tear n=21	Average Pain Complete Tear n=11	p-value
				0 months	8.10 ± 0.83	7.81 ± 0.98	0.406
3 months	3.81 ± 3.04	4.36 ± 3.50	0.645
6 months	3.95 ± 2.85	5.27 ± 3.55	0.262
12 months	4.81 ± 2.64	5.55 ± 3.21	0.492

Regarding average pain variation for 12 months, Table 7 and Table 8 compare both ‘partial tear’ and ‘complete tear’ groups, respectively. Similarly, to previous groups, there was a statistically significant decrease in mean pain in both groups when comparing any period of evaluation to baseline. In the ‘Partial Tear’ group, variations beyond three months were not statistically significant. On the other hand, in the ‘Complete Tear’ group, there was an increase in average pain between three and 12 months (p=0.046).

**Table 7:** Average pain comparison between different evaluation periods in the ‘Partial Tear’ group The first column indicates the beginning (X) and the end of the same period (Y). The second and third columns show the mean and standard deviation of pain in periods X and Y, respectively. The fourth column shows pain variation between periods X and Y. The fifth column represents the statistical difference (p-value).

‘Partial Tear’ Group n=20 period X to Y	Average Pain X	Average Pain Y	Variation (Y-X)	p-value
					0 to 3 months	8.10 ± 0.83	3.81 ± 3.04	- 4.28 ± 3.35	<0.001
0 to 6 months	8.10 ± 0.83	3.95 ± 2.85	- 4.14 ± 3.14	<0.001
0 to 12 months	8.10 ± 0.83	4.81 ± 2.64	- 3.29 ± 2.78	<0.001
3 to 6 months	3.81 ± 3.04	3.95 ± 2.85	+ 1.43 ± 2.33	0.782
3 to 12 months	3.81 ± 3.04	4.81 ± 2.64	+ 1.00 ± 3.22	0.171
6 to 12 months	3.95 ± 2.85	4.81 ± 2.64	+ 0.86 ± 2.08	0.74

**Table 8:** Average pain comparison between different evaluation periods in the ‘Full Tear’ group The first column indicates the beginning (X) and the end of the same period (Y). The second and third columns show the mean and standard deviation of pain in periods X and Y, respectively. The fourth column shows pain variation between periods X and Y. The fifth column represents the statistical difference (p-value).

‘Complete Tear’ Group n=12 (period X to Y)	Average Pain X	Average Pain Y	Variation (Y-X)	p-value
					0 to 3 months	7.81 ± 0.98	4.36 ± 3.50	- 3.45 ± 2.94	0.003
0 to 6 months	7.81 ± 0.98	5.27 ± 3.55	- 2.55 ± 2.98	0.018
0 to 12 months	7.81 ± 0.98	5.55 ± 3.21	- 2.27 ± 2.80	0.022
3 to 6 months	4.36 ± 3.50	5.27 ± 3.55	+ 0.91 ± 2.63	0.277
3 to 12 months	4.36 ± 3.50	5.55 ± 3.21	+ 1.18 ± 1.72	0.046
6 to 12 months	5.27 ± 3.55	5.55 ± 3.21	+ 0.27 ± 2.53	0.728

**Figure 3:**
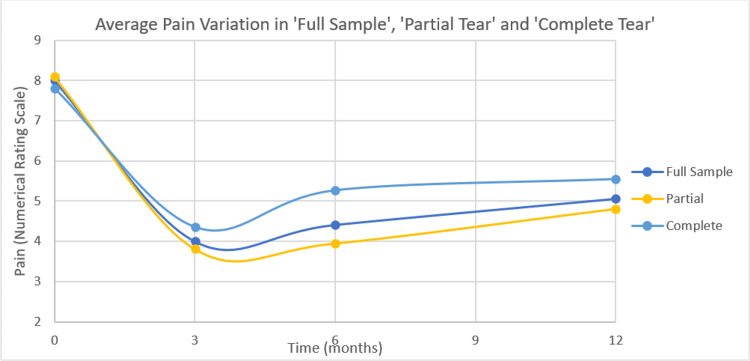
Average pain variation in ‘Full Sample’, ‘Partial Tear’ and ‘Complete Tear’ groups

Figure 3 graphically represents variation between baseline, three months, six months, and 12 months in both the ‘Partial Tear’ and ‘Complete Tear’ groups, as well as in ‘Full Sample’.

Adverse effects occurrence

Regarding adverse effects occurrence, only one minor event was reported (3%). A 61-year-old male, without previous known pathology, had a vasovagal syncope. The patient returned to normal status after a few minutes of lying down with leg elevation.

## Discussion

According to our results, suprascapular nerve pulsed radiofrequency decreased average pain at least 36% for 12 months, with a 50% peak pain reduction at three months after the procedure. Average pain increased by 26% between three and 12 months after the procedure, however, it was always significantly inferior, compared to baseline. These results suggest that suprascapular nerve pulsed radiofrequency is effective in reducing pain at 12 months in patients with supraspinatus tears, with a peak pain reduction effect at three months, and a slight slow fading of the pain reduction in the following nine months. Our results are similar to other authors where suprascapular nerve pulsed radiofrequency reduced shoulder chronic pain by approximately 50% at three months and 35% at six months [18]. Nonetheless, suprascapular pulsed radiofrequency might be even more effective in relieving pain in other shoulder conditions, such as hemiplegic shoulder pain, where it might achieve an average pain reduction at three months of 60% [19], and frozen shoulder, with a 74% average pain reduction at three months [20].

Our analysis suggests that cortico-anaesthetic intra-articular shoulder injections (80 mg of methylprednisolone and 30mg of lidocaine) do not add any benefit in shoulder pain reduction when simultaneously performed with suprascapular nerve pulsed radiofrequency. As previously mentioned, simultaneously with pulsed radiofrequency, a group of patients was also submitted to an intra-articular shoulder injection. We, therefore, analyzed both groups independently. Consulting clinical records, a clear reason for adding intra-articular injection to pulsed radiofrequency was not clear. In fact, there was no difference between average baseline pain for both groups. We infer that the physician's decision was of personal preference. We also compared average pain in both groups at three, six and 12 months and no statistically significant difference was found. These results suggest that intra-articular cortico-anaesthetic injection, simultaneously to suprascapular nerve pulsed radiofrequency, doesn’t add any pain relief benefit. Compared to baseline, both groups had a significant pain reduction at three, six and 12 months. Isolated pulsed radiofrequency group had 44% pain reduction at 12 months, compared to 25% in intra-articular injection plus pulsed radiofrequency group. However, as stated, this difference was not of statistical significance.

Some authors describe partial tears as more painful than full-thickness tears [10]. Even though the differences found between our ‘partial tear’ and ‘complete tear’ groups were not statistically significant, our findings resemble those of the Wolff et al. study, with average pain of 8.10 out of 10 on the numerical rating scale in ‘partial tear’ group compared with 7.81 out of 10 in ‘complete tear’ group. Once again, comparing both groups at three, six and 12 months, there was no statistical difference found, suggesting that either partial or complete supraspinatus tear pain improves with pulsed radiofrequency. In the ‘partial tear’ group, average pain decreased more than 50% at three months (p<0.001), increasing 26% from three to 12 months (p=0.171). On the other hand, in the ‘complete tear’ group, average pain decreased 44% at three months (p=0.003), increasing 27% from three to 12 months (p=0.046). Even though there were no differences between groups at three, six and 12 months, these results suggest that complete tears might have a smaller pain peak reduction at three months, with a significant increase overtime. Nonetheless, average pain reduction after the procedure is always statistically significant compared to baseline, either in partial or complete tears.

The mechanism of action behind suprascapular nerve pulsed radiofrequency pain decreasing efficiency is not fully understood, but its cornerstone might be neuromodulation. Pulsed radiofrequency uses radiofrequency in short (20ms), high-voltage bursts, with an ON: OFF ratio of 20: 480ms, allowing heat to be eliminated [13]. Early studies suggested a molecular pathway involving the *c-Fos* gene (an immediate early gene) [15] or ATF3 (cellular stress indicator) [16] as a mechanism of action. Recent evidence shows that non-pharmacological interventions, such as neuromodulation techniques, have the potential to counteract maladaptive neuroplasticity, alleviate chronic pain, and might even prevent acute-to-chronic pain transition [21]. Our results suggest that suprascapular nerve pulsed radiofrequency probably acts via peripheral molecular mechanisms of action, leading to a peak pain reduction at three months, with subsequent pain chronification mechanism interference, reducing shoulder pain for, at least, 12 months.

As stated before, this study took place in a reference Rehabilitation Centre Musculoskeletal Rehabilitation and Intervention Unit, where tertiary care should be provided. Even though clinical records were not clear if either the patients previously underwent classic rehabilitation treatments, such as physiotherapy, we expect that minimally invasive treatment options were only pursued after conservative management failed. However, our analysis evidenced that proper analgesic pain management is lacking in these patients, as only 13% of patients were prescribed daily scheduled analgesic drugs and only 25% were prescribed with on-demand pain relievers, despite having severe pain, by numerical rating scale, at baseline. Nonetheless, future studies should analyze the cost-effectiveness of thoroughly purse conservative management, compared to early minimal invasive interventions.

Even though our results are promising in highlighting suprascapular nerve pulsed radiofrequency role in supraspinatus tears pain management, we must list some possible biases present in our study: 1) first, our analysis was retrospective. However, one should consider that the capability of designing a double-blinded randomized controlled trial, either logistically or ethically, is doubtful. On one hand, pulsed radiofrequency execution relies on physician technique and patient feedback, making it hard to be double-blinded. On the other hand, our results seem to point in a way where a placebo would not be ethically viable, as the patients significantly improved in pain numerical rating scale with pulsed radiofrequency; 2) as our study was retrospective, we resorted to clinical records as the main source of information, and any missing variable was obtained through a phone call with the patient. Thus, the possibility of information bias is present; 3) our sample included 32 patients which, due to the prevalence of the pathology studied, could be seen as suboptimal. Future studies should be pursued, focusing on prospective study designs, with larger samples and, besides pain evaluation, shoulder functionality and quality of life scores should be determined.

## Conclusions

Our retrospective analysis revealed average shoulder pain reduction for, at least, 12 months following suprascapular nerve pulsed radiofrequency, with a peak pain reduction at three months. Slow fading of pain reduction in the following nine months was seen, however, compared to baseline, pain reduction was always statistically significant. Furthermore, our results suggest that cortico-anaesthetic intra-articular shoulder injections do not add benefit in shoulder pain reduction, simultaneously with SNpRF.

We identified poor pharmacological analgesic management, either scheduled or on demand, as a pitfall in this population. Further efforts should be made, not only in primary and secondary lines of care but also in reference centers, to explore the full potential of conservative management. Only after exhausting analgesic medication management, as well as classic physiatric treatment, should minimal invasive intervention be pursued. Future studies should also analyze the cost-effectiveness between these two lines of acting. Moreover, despite our promising results to fully understand pulsed radiofrequency long-term benefit in shoulder pain, larger prospective studies, analyzing shoulder functionality and quality of life lost scores, besides pain reduction, should be pursued.
